# Physiological and biochemical responses of *Ricinus communis* seedlings to different temperatures: a metabolomics approach

**DOI:** 10.1186/s12870-014-0223-5

**Published:** 2014-08-12

**Authors:** Paulo Roberto Ribeiro, Luzimar Gonzaga Fernandez, Renato Delmondez de Castro, Wilco Ligterink, Henk WM Hilhorst

**Affiliations:** 1 Wageningen Seed Lab, Laboratory of Plant Physiology, Wageningen University, Droevendaalsesteeg 1, Wageningen, 6708 PB, The Netherlands; 2 Laboratório de Bioquímica, Biotecnologia e Bioprodutos, Departmento de Biofunção, Universidade Federal da Bahia, Reitor Miguel Calmon s/n, Salvador, 40160-100, Brazil

**Keywords:** Carbon-nitrogen balance, Castor bean, Ricinus communis, Seedling establishment, Temperature

## Abstract

**Background:**

Compared with major crops, growth and development of *Ricinus communis* is still poorly understood. A better understanding of the biochemical and physiological aspects of germination and seedling growth is crucial for the breeding of high yielding varieties adapted to various growing environments. In this context, we analysed the effect of temperature on growth of young *R. communis* seedlings and we measured primary and secondary metabolites in roots and cotyledons. Three genotypes, recommended to small family farms as cash crop, were used in this study.

**Results:**

Seedling biomass was strongly affected by the temperature, with the lowest total biomass observed at 20°C. The response in terms of biomass production for the genotype MPA11 was clearly different from the other two genotypes: genotype MPA11 produced heavier seedlings at all temperatures but the root biomass of this genotype decreased with increasing temperature, reaching the lowest value at 35°C. In contrast, root biomass of genotypes MPB01 and IAC80 was not affected by temperature, suggesting that the roots of these genotypes are less sensitive to changes in temperature. In addition, an increasing temperature decreased the root to shoot ratio, which suggests that biomass allocation between below- and above ground parts of the plants was strongly affected by the temperature. Carbohydrate contents were reduced in response to increasing temperature in both roots and cotyledons, whereas amino acids accumulated to higher contents. Our results show that a specific balance between amino acids, carbohydrates and organic acids in the cotyledons and roots seems to be an important trait for faster and more efficient growth of genotype MPA11.

**Conclusions:**

An increase in temperature triggers the mobilization of carbohydrates to support the preferred growth of the aerial parts, at the expense of the roots. A shift in the carbon-nitrogen metabolism towards the accumulation of nitrogen-containing compounds seems to be the main biochemical response to support growth at higher temperatures. The biochemical changes observed in response to the increasing temperature provide leads into understanding plant adaptation to harsh environmental conditions, which will be very helpful in developing strategies for *R. communis* crop improvement research.

## Background

Castor bean (*Ricinus communis* L.) is a member of the Euphorbiaceae and can be found throughout tropical, sub-tropical and warm temperate regions [1],[2]. Castor oil has been primarily used as purgative or laxative in traditional medicine to counter constipation [2], but the commercial interest in Castor bean is mainly increasing because its seeds contain high amounts of a unique oil consisting of up to 94% of the fatty acid ricinoleic acid (12-hydroxy-cis-9-octadecenoic acid) [3]. This fatty acid confers unique and highly demanded properties to the oil and the biodiesel produced from it: its high calorific value and the high cetane number are of advantage along with the low content of phosphorous and carbon residues [4]-[7]. It is at present one of the few commercially available sources of natural hydroxylated triglycerides [8].

The Brazilian National Program for Production and Use of Biodiesel has identified *R. communis* as the ideal oil crop to promote social development in the semi-arid region of Brazil because of its versatility as a productive (oil) crop in various environments [9]. For this reason *R. communis* is currently grown in the arid zones of Northeastern Brazil [10]. Genotypes MPA11, MPB01 and IAC80 were developed by the breeding program of the Campinas Agronomic Institute and by the *Empresa Baiana de Desenvolvimento Agrícola S.A* (EBDA), aiming at finding alternative high yielding crops for family farmers in the semi-arid region of Brazil.

Temperature is a critical environmental cue that influences seedling establishment and a difference of only a few degrees may already lead to a notable change in growth and survival rate [11]. The effects of the temperature on the survival, growth, photosynthesis, and metabolism of young seedlings has been assessed for several plants [12]-[15], but growth and development of Castor bean is still poorly understood, as compared with major crops. Therefore, a better understanding of the biochemical and physiological aspects of germination and seedling growth is crucial for the breeding of high yielding varieties adapted to various growing environments that could be used for family farmers worldwide [16].

Metabolite profiling of plants under different environmental conditions has provided important information about the biochemical and molecular changes related to temperature adaptation. Carbohydrate and amino acid metabolism appear to be part of the mechanisms by which plants adapt to different environmental conditions [17]-[20]. Temperature may also affect heat tolerance in terms of seedling growth, antioxidant response and cell death [21], whilst in general, plants display considerable plasticity to respond to short-term fluctuations of environmental factors [22]-[24]. Although metabolomics has been used to dissect plant responses to abiotic stresses, most of the studies regarding the temperature effect on seedling performance have focused on the ability of plants to maintain homeostasis at chilling temperatures (0 to 15°C) or have investigated plant responses to high-temperature stress, mostly using Arabidopsis as model species [25]-[27]. Plant metabolic plasticity in response to mild temperatures (20 to 35°C) has received much less attention although it is an important trait for crop species [28]. *R. communis* possesses the ability to grow in various diverse environments which makes this species an ideal candidate to provide a better understanding of seedling performance and adaptation under different temperatures.

Initial vegetative growth is very important to the establishment of Castor bean and since little is known about biochemical and molecular changes related to temperature adaptation in *R. communis*, we raise the question whether such a highly adaptable species may have a specific metabolic signature that may apply to other crops, as well. In this context, we set out to study the Castor bean metabolome in young seedlings and its relationship with seedling growth performance at different temperatures.

## Methods

### Plant material

Seeds of three different *Ricinus communis* genotypes (IAC80, MPA11 and MPB01) used in this work were kindly supplied by “*Empresa Baiana de Desenvolvimento Agrícola S.A.*” (EBDA), Salvador-Bahia, Brazil. The genotype IAC80 was developed by the breeding program of the Campinas Agronomic Institute aiming to recommend it to small family farms that use manpower to carry out the harvest in several steps. This genotype enables high yields of *R. communis* seeds concomitantly with the production of food crops. Genotypes MPA11 and MPB01 were bred by *Empresa Baiana de Desenvolvimento Agrícola S.A* (EBDA) aiming at finding alternative crops for family farmers at the semi-arid region of Brazil.

For phenotypical assays, seeds were allowed to germinate using paper rolls as substrate at 25°C in the dark. After 72 hours, germinated seeds were transferred to moist soil and were allowed to grow at five different temperatures (20°C, 25°C, 30°C and 35°C) in continuous light for 13 days. Fresh and dry weight of the cotyledons, first true leaves, cotyledons, stem and roots of the 14-day-old seedlings were measured together with stem height. Fifteen to twenty seedlings were used.

For metabolite profiling assays, seed coats were removed and the seeds were allowed to germinate using paper rolls as substrate at 25°C in the dark. After 44–50 hours, germinated seeds were transferred to moist vermiculite and were allowed to grow at 20°C in continuous light for 10 days. This was done to reduce differences in developmental stages that were observed during the phenotypical characterization when plants grew over a period of 13 days in different temperatures. Then, half of the 10-day-old seedlings were then transferred to an incubator at 35°C with continuous light. After 4 days, roots and cotyledons (three biological replicates of 15–18 seedlings each) were collected, immediately frozen in liquid nitrogen, freeze-dried, ground and stored at −80°C prior to analysis.

### Extraction and derivatization of primary metabolites for GC-TOF-MS analysis

Metabolite profiling analysis was performed as described previously [29]. Approximately 20 mg of freeze-dried and ground roots or cotyledons were transferred to a 2-mL Eppendorf tube after which 400 μL methanol and 300 μL chloroform were added and vortexed briefly. Then, 130 μL of H_2_O and 20 μL of the internal standard ribitol (1 mg/mL) were added, vortexed thoroughly and sonicated for 10 minutes. Thereafter, 200 μL of H_2_O was added, vortexed thoroughly and centrifuged for 5 min at 17000 g in an Eppendorf centrifuge. Of the upper phase 600 μL were transferred to a new 2-mL Eppendorf tube. To the remaining material 500 μL of a 1:1 v/v mix of methanol and chloroform was added, vortexed thoroughly and kept on ice for 10 minutes. Then, 200 μL of H_2_O was added and centrifuged for 5 min at 17000 g in an Eppendorf centrifuge. Of the upper phase 400 μL were transferred to the previously collected upper phase. An aliquot of 30 μL of the joint upper phase was dried overnight by vacuum centrifugation.

All samples were analyzed by gas chromatography coupled to a quadrupole time of flight mass spectrometry system (GC-TOF-MS) as TMS derivatives. TMS derivatives were obtained by online derivatization (Combi PAL autosampler - CTC Analytics) as described previously [30]. Initially, 12.5 μL of O-methylhydroxylamine hydrochloride (20 mg mL^−1^ in pyridine) was added to the vials and incubated for 30 min at 40°C in a shaking incubator. Then, 17.5 μL of N-methyl-N-trimethylsilyltrifluoroacetamide (MSTFA) was added to the vials for 60 min at 40°C in a shaking incubator. 5 μL of an alkane mixture (C10–C17 and C19–C33) was added to determine the retention index of the metabolites. Sample aliquots of 2 μL were injected at a split ratio of 20:1 into an Optic 3 high-performance injector (ATAS) at 70°C, and then the injector was rapidly heated to 240°C at 6°C s^−1^. Chromatography was performed in an Agilent 6890 gas chromatograph (Agilent Technologies) coupled to a Pegasus III time-of-flight mass spectrometer (Leco Instruments) using a VF-5 ms capillary column (Varian; 30 m × 0.25 mm × 0.25 μm) including a 10-m guardian column with helium as carrier gas at a column flow rate of 1 mL min^−1^. The oven temperature program was 2 min at 70°C, followed by a 10°C min^−1^ ramp to 310°C, 5 min at 310°C, and 6 min at 70°C before the next injection. The transfer line temperature was set at 270°C. The column effluent was ionized by electron impact at 70 eV. Mass spectra were recorded at 20 scans s^−1^ within a mass-to-charge ratio range of 50 to 600 at a source temperature of 200°C. A solvent delay of 295 s was set. The detector voltage was set to 1,650 V.

### Extraction of secondary metabolites for GC-MS analysis

Approximately 10 mg of freeze-dried and ground roots or cotyledons were transferred to a 2- mL Eppendorf tube and 300 μL or 150 μL of a methanol:chloroform (1:1) solution was added, respectively. Subsequently, the tubes were sonicated for 10 minutes and centrifuged for 5 min at 17000 g in an Eppendorf centrifuge. The upper phase (100 μL) was filtered and transferred to a 2-mL glass GC vial with a 200-μL glass insert. Residual water was removed with sodium sulfate during filtration. Hexadecane and heptadecane (1:1) in hexane were used as internal standards.

Chromatography was performed with an Agilent 7809A gas chromatograph (Agilent Technologies) coupled to a Triple-Axis detector (Agilent 5975C), using a ZB-5 (Phenomenex; 30 m × 0.25 mm) capillary column (0.25 mm film thickness) using helium as carrier gas at a flow rate of 1 mL min^−1^. Three different split ratios were used: 29:1 with detector off between 18.8 and 19.2 min for *R. communis* green cotyledon samples, 9:1 for *R. communis* root samples and 149:1 during ricinine quantification. The inlet temperature of the injector was set to 250°C. The initial oven temperature was 45°C for 1 min, and was increased to 300°C after 1 min at a rate of 10°C min^−1^ and held for 5 min at 300°C. A solvent delay of 240 s was set.

### Extraction of soluble carbohydrates and starch

Soluble carbohydrates were determined as described by Bentsink [31], with minor modifications. Fifteen milligrams of freeze-dried and ground roots or cotyledons were transferred to a 2-mL Eppendorf tube and homogenized in 1 mL of methanol (80% v/v) with the addition of 10 μg of lactose as internal standard. Samples were incubated in a water bath for 15 minutes at 76°C. Samples were completely dried by vacuum centrifugation. Then, 500 μL of milliQ water was added, thoroughly vortexed and centrifuged for 5 min at 17000 g in an Eppendorf centrifuge. The supernatant was injected into a Dionex HPLC system (Dionex, Sunnyvale, CA) to analyze soluble carbohydrates, using a CarboPac PA100 4- × 250-mm column (Dionex) preceded by a guard column (CarboPac PA100, 4 × 50 mm), a gradient pump module (model GP40, Dionex) and an ED40-pulsed electrochemical detector (Dionex). Mono-, di-, and trisaccharides were separated by elution in an increasing concentration of NaOH (50–200 mM) with a flow rate of 1 mL per minute. Peaks were identified by co-elution of standards. Sugar quantity was corrected by means of the internal standard and transformed to micrograms of sugar per milligram of dry material.

Starch was determined as glucose, using an EnzyPlus glucose test kit (BioControl, art.nr. EZS 781+), based on NADPH + H^+^ formation that is measured with a spectrophotometer at 340 nm. The remaining pellets from the previous carbohydrate analysis were used to quantify starch. Pellets were washed 3× with milliQ water in order to remove all remaining soluble carbohydrates. Samples were completely dried by vacuum centrifugation after the final washing step. Ten mg of dried material were weighed and homogenized with 50 μL 8 N HCl and 200 μL DMSO. Samples were incubated in a water bath for 60 minutes at 60°C. After this 300 μL milliQ water and 80 μL 5 N NaOH were added, vortexed immediately, followed by addition of 370 μL citric acid (pH 4.60), thoroughly vortexed and centrifuged for 1 min at 17000 g. The clear supernatant was used for starch determination according to the instructions provided with the kit.

### GC-MS data processing and compound identification

Initially, raw data were first processed by ChromaTOF software 2.0 (Leco Instruments), and further baseline correction, accurate mass calculation, data smoothing and noise reduction, followed by alignment between chromatograms were performed using the MetAlign software [32]. MSClust was used to remove metabolite signal redundancy in aligned mass peaks tables and to retrieve mass spectral information of metabolites using mass peak clustering [33]. This resulted in a set of reconstructed metabolites (representative masses). The mass spectra of the representative masses were used for tentative identification by matching to the spectral libraries (National Institute of Standards and Technology [NIST08]; Golm metabolome database [http://gmd.mpimp-golm.mpg.de/]) and by comparison of the retention index calculated using a series of alkanes. Authentic reference standards were used to confirm the identity of the metabolites. Levels of identification are presented at Additional file 1: Table S1 and Additional file 2: Table S2 according to Summer [30].

### Multivariate statistical analysis

Mass intensity values of the representative masses were normalized by the internal standard and by the weight. Normalized and log transformed data were subjected to statistical analysis using Canoco 5 (http://www.canoco5.com/). In order to compare the overall variation in metabolite composition explained by differences in temperature, tissue and genotype, as well as to evaluate the importance of individual axes of constrained (Redundancy Analysis - RDA) and unconstrained ordination (Principal Component Analysis - PCA), we performed a “compared-constrained-unconstrained” analysis in Canoco 5. Temperature, tissue and genotype were used as explanatory variables.

Normalized data were also uploaded at MetaboAnalyst 2.0, a web-based analytical pipeline for high-throughput metabolomics studies (http://www.metaboanalyst.ca/MetaboAnalyst/). Row-wise normalization was performed to allow general-purpose adjustment for differences among samples. Log transformation and auto-scaling were performed to make features more comparable. Uni- and multivariate analysis were performed using log transformed and auto-scaled data. Metabolite-metabolite correlation analysis was also performed on the entire data set of metabolites using Pearson’s correlation.

## Results

### Growth and biomass production are strongly affected by environmental conditions in *Ricinus communis* seedlings

**Figure 1:**
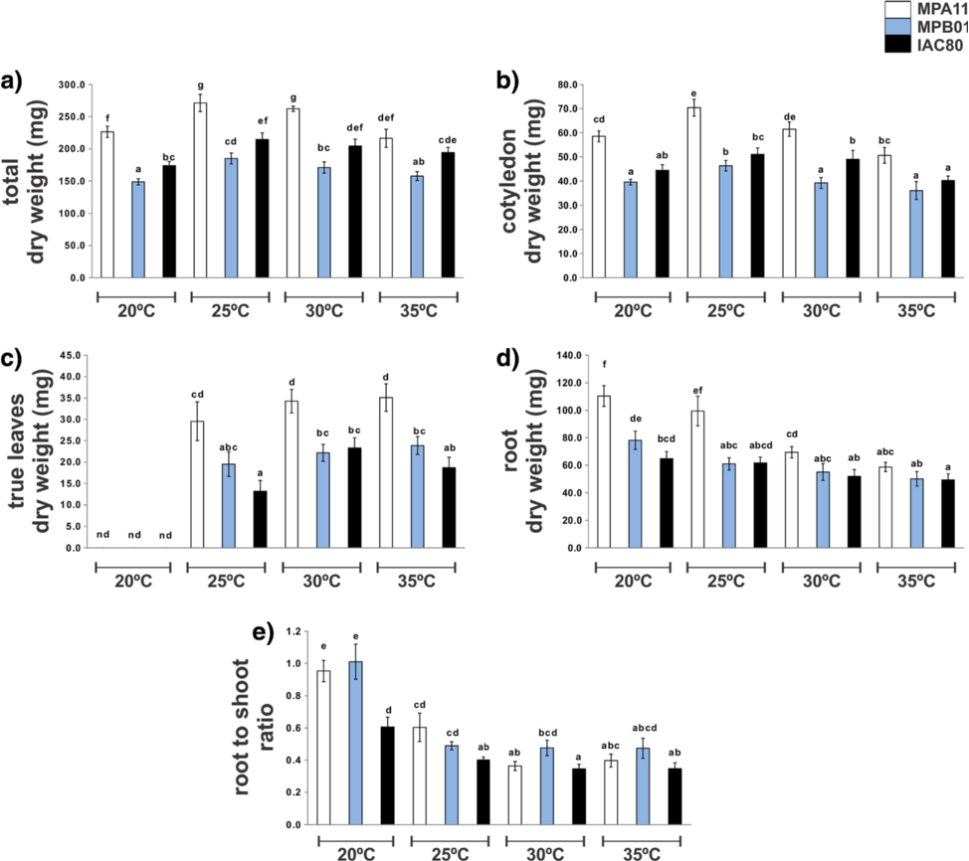
***R. communis*seedling growth performance.** Total **(a)**, cotyledon **(b)**, first true leaves **(c)** and roots **(d)** dry weight and root to shoot ratio **(e)** of 14-day-old seedlings of three different genotypes, growing at 4 different temperatures (20, 25, 30 and 35°C). Results for genotypes MPA11 (white bars), MPB01 (blue bars) and IAC80 (black bars) are shown.

In order to explore the effect of temperature on *R. communis* seedling growth attributes we measured the dry weight of cotyledons, true leaves and roots of 14-day-old seedlings, grown at 4 different temperatures (20, 25, 30 and 35°C) (Figure 1). For this purposes we used three different genotypes: MPA11, MPB01 and IAC80. The genotype MPA11 displayed a greater dry biomass which makes it clearly different from the other two genotypes. The lowest total biomass of the genotype MPA11 was observed at 20 and 35°C and the highest at 25 and 30°C. For genotypes MPB01 and IAC80 the lowest total biomass was observed at 20°C and no differences in total biomass were observed between seedlings grown at 25, 30 and 35°C (Figure 1a).

The cotyledon biomass for the genotype MPB01 and IAC80 showed minor changes in response to the temperature, while for genotype MPA11 the highest cotyledon biomass was observed at 25 and 30°C (Figure 1b). The first pair of true leaves of seedlings growing at 20°C could not develop and consequently the 14-day-old seedlings showed no pair of true leaves. The dry weight of the first pair of true leaves of the genotypes MPA11 and MPB01 was not significantly affected by the temperature, and for genotype IAC80 growing at 30°C the first pair of true leaves was slightly heavier that those growing at 25 and 35°C (Figure 1c).

For genotype MPA11, root biomass decreased with increasing temperature reaching the lowest value at 35°C (Figure 1d). The root weight was negatively influenced by the temperature, apparently to the benefit of the aboveground part. On the other hand, root biomass of genotypes MPB01 and IAC80 was not appreciably affected by the temperature suggesting that the root of these genotypes are less sensitive to the temperature (Figure 1d). However, the decrease of the root to shoot ratio with temperature was consistent and appeared to be independent of the genotype (Figure 1e).

### Changes in primary metabolite content in root and cotyledons in response to two different growth temperatures

In order to unravel metabolic changes associated with the temperature during seedling establishment we measured metabolite levels in roots and cotyledons of young seedlings growing at 20 and 35°C. We detected over 100 peaks in root samples, of which 54 could be annotated, while in cotyledons samples we detected over 200 peaks of which 69 were annotated (Additional file 1: Table S1). Initially, redundancy analysis (RDA) was used to compare the overall variation in metabolite composition associated with temperature, tissue and genotype differences (Additional file 3: Figure S1). Principal component 1 discriminated root and cotyledon samples explaining nearly 94% of the total variance. Principal component 2 separated samples that were grown at 20 and 35°C and explained only 2% of the total variance. The RDA plot shows that most of the variation in the metabolite composition must be attributed to differences between the tissues rather than between temperatures. Therefore, in order to assess whether the temperature has an effect on the metabolite composition of a given tissue, we decided to analyze cotyledon and root samples separately.

**Figure 2:**
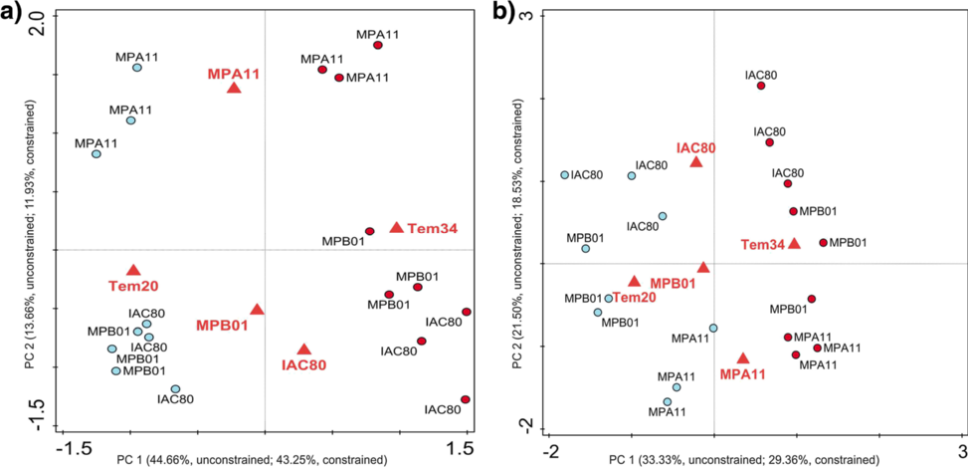
**Redundancy analysis based on polar metabolite profiles in response to an increase in temperature.** RDA plot in the cotyledons **(a)** and roots **(b)** based on polar metabolite profiles. The distance between genotypes approximates the average dissimilarity of metabolite composition between the two sample classes being compared as measured by their Euclidean distance, whereas the distance between replicates approximates the dissimilarity of their metabolite content as measured by their Euclidean distance. The distance of selected sample symbol (circles) from temperature and genotypes symbols (triangles) predicts the sample membership in one of the classes. It can also be seen as the dissimilarity between metabolite composition of that sample and average metabolite composition of samples belonging to individual classes. Score scaling is focused on standardized metabolites scores.

When analyzed separately, for both cotyledon and root, principal component 1 discriminated samples that were grown at 20 and 35°Cwhile principal component 2 discriminated the natural variance contribution of the different genotypes. Based on the RDA analysis, genotypes MPB01 and IAC80 are closely related, whereas genotype MPA11 differed more in terms of metabolome. However, just a few metabolites appear to be responsible for this difference (Figure 2, Additional file 4: Table S3).

#### Overall changes in amino acid content in response to an increase in temperature

**Figure 3:**
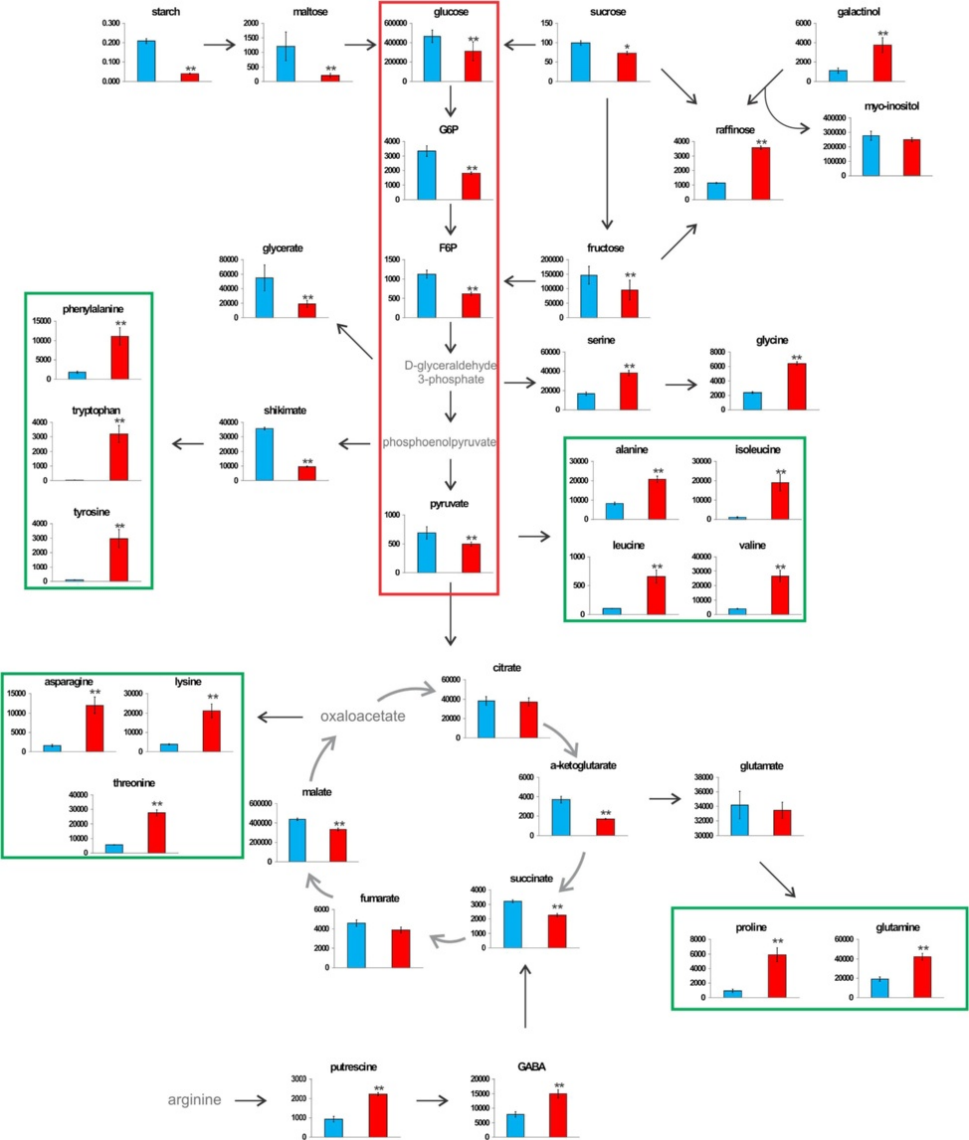
**Mapping of relative metabolite concentrations on known pathways for cotyledons of*Ricinus communis*genotype MPA11.** Seedlings were grown at 20°C (blue columns) or at 35°C (red columns). Data means and standard errors of three biological replicates containing 15–18 seedlings each are shown. Metabolites in grey (without column graphs) were not detected. Sucrose content was determined by HPLC. *p < 0.05 **FDR < 0.01.

**Figure 4:**
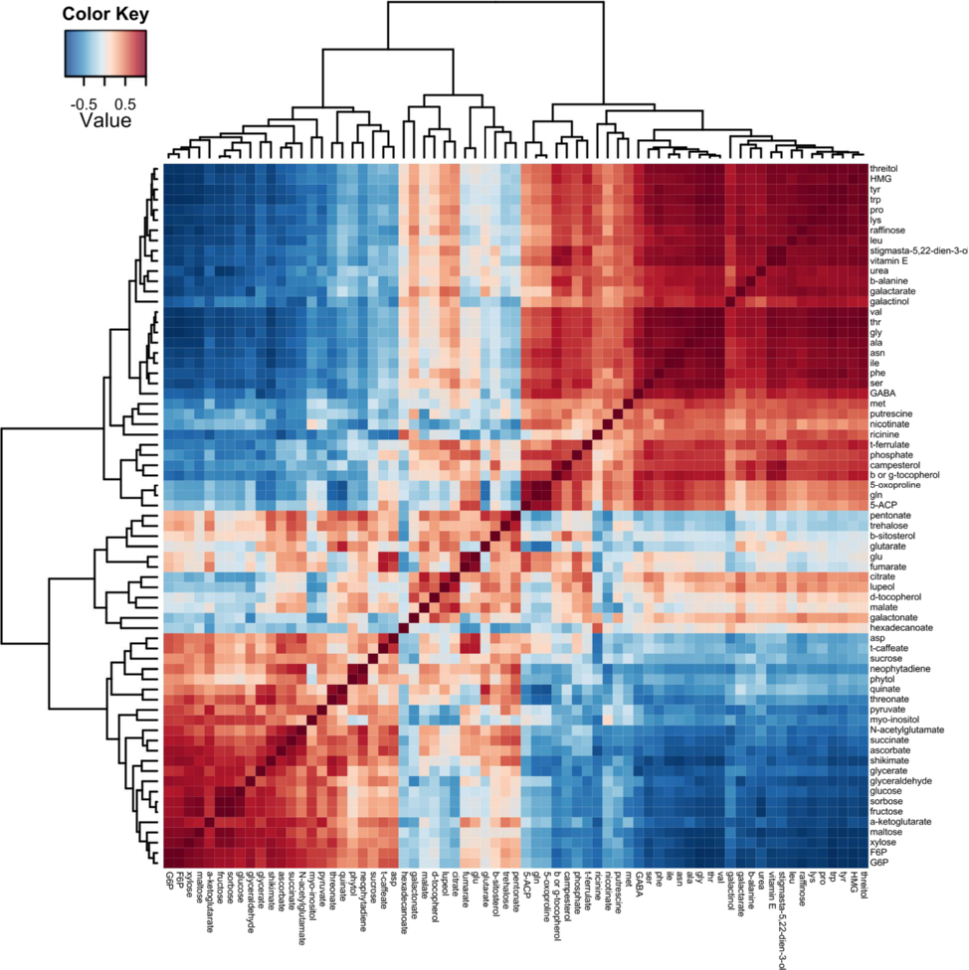
**Hierarchical cluster analysis.** Heatmap representation of the metabolite-metabolite correlations in response to the temperature treatment in cotyledon samples of three *R. communis* genotypes. Correlations coefficients were calculated based on Pearson’s correlation.

A total of 21 and 14 amino acids were identified in cotyledons and roots, respectively (Additional file 1: Table S1). In the RDA plots, they are strongly associated with the temperature of 35°C, which is in agreement with the fact that the levels of almost all identified amino acids increased at 35°C (Figure 3; Additional file 5: Figure S2). In the cotyledons, most of the amino acid increased 2 to 10-fold at 35°C, however methionine, tyrosine and tryptophan showed a steeper increase, varying from 20 to 220 fold change. Statistically significant differences in methionine levels were only observed in the genotype IAC80 (53-fold) (Additional file 6: Table S4). Although glutamate content did not vary significantly, one of its biosynthetic derivatives, 4-aminobutyric acid (GABA) showed significant changes related to the temperature (FDR < 0.01). GABA increased 1.9-fold at 35°C for genotype IAC80 (Additional file 1: Tables S1 and Additional file 6: Table S4). Amino acids were strongly and positively correlated with each other as well as with some sugars (raffinose and galactinol) and weakly correlated with some organic acids (fumarate and citrate). In addition, strong positive correlations were found for *β-*alanine, putrescine, vitamin E and other tocopherols, urea, 3-hydroxy-3-methylglutarate and ricinine (Figure 4).

In the roots, much less variation in amino acid content was found (FDR < 0.01) in response to the temperature. Only 6 out of 14 of the detected amino acids were affected by the temperature (FDR < 0.01), but to a lesser extent than in cotyledons (Additional file 1: Table S1). Amino acids were strongly and positively correlated with each other and weakly correlated with some organic acids (fumarate and citrate). In addition, strong positive correlations were found with vitamin E and other tocopherols, urea, 3-hydroxy-3-methylglutarate and ricinine (Additional file 7: Figure S3).

#### Overall carbohydrate changes in response to an increase in temperature

The initial reduction in the levels of starch in cotyledons (up to 5.3 fold) in response to the increasing temperature (Figure 3) suggests that as soon as the seedlings are transferred to higher temperatures, carbohydrate mobilization and metabolism is up-regulated. In the roots, no starch could be detected. In both cotyledons and roots, RDA shows that carbohydrates are strongly associated with the temperature of 20°C, which is in agreement with the fact that the levels of the majority of identified carbohydrates were higher at 20°C (Figures 3; Additional file 5: Figure S2). Glucose, fructose, galactose, sorbose, sucrose and xylose levels decreased in cotyledons (1.3 to 3-fold), but to a much greater extent in the roots (5.8 to 20 times) at 35°C (Additional file 1: Table S1). These carbohydrates were negatively correlated with amino acids, suggesting that a shift in the carbon-nitrogen metabolism occurs in response to the temperature change (Figure 4; Additional file 7: Figure S3). Interestingly, maltose was the only carbohydrate that decreased more in the cotyledons than in the roots at 35°C. Raffinose and galactinol were the only carbohydrates that increased (up to 4-fold) at 35°C in cotyledons and roots as compared to 20°C (Additional file 1: Table S1) and they were negatively correlated with the others carbohydrates (Figure 4; Additional file 7: Figure S3).

#### Changes in tricarboxylic acid cycle (TCA) and glycolytic intermediates in response to an increase in temperature

Glycolysis and the TCA cycle are key metabolic routes by which organisms generate energy from carbohydrates, amino acids and fatty acids. TCA cycle and glycolytic intermediates were found to be affected by temperature (FDR < 0.01) and RDA suggests a close relationship among these compounds (Figures 3 and 4; Additional file 7: Figure S3). The glycolytic intermediates glucose-6-phosphate (G6P), fructose-6-phosphate and pyruvate were up to 3-fold reduced in the cotyledons of seedlings grown at 35°C, but only G6P was detected in the roots and showed the same behavior as in the cotyledons. In both tissues, four TCA cycle intermediates were detected: fumarate, citrate, succinate and malate and α-ketoglutarate was detected in the cotyledons only. All TCA cycle intermediates detected in the roots showed a decrease (from 1.5 to 3.5-fold) at 35°C; however, in the cotyledons only α-ketoglutarate and succinate showed the same behavior. Fumarate, citrate and malate were slightly increased in the cotyledons (2-fold) at 35°C (Figure 3; Additional file 1: Table S1). Metabolite-metabolite correlations of amino acids, glycolytic and TCA cycle intermediates confirm the strong relationship between these metabolic pathways (Figure 4).

### Changes in contents of secondary metabolites in response to temperature increase

**Figure 5:**
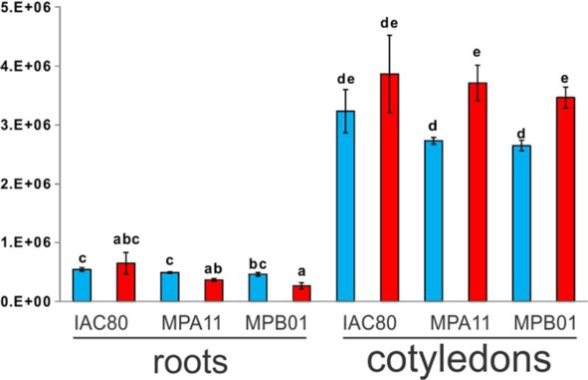
**Relative ricinine levels in root and cotyledon samples of*Ricinus communis*.** Seedlings were grown at 20°C (blue columns) or at 35°C (red columns). Data means and standard errors of three biological replicates containing 15–18 seedlings each are shown.

As an increase in ricinine content of growing Ricinus seedlings had been reported [34], we decided to assess the effect of temperature on the relative amounts of ricinine and other secondary metabolites in roots and cotyledons of young seedlings. We detected over 100 peaks in root samples and over 50 peaks in cotyledon samples. Unfortunately, we could identify only 15 and 12 metabolites in roots and cotyledons, respectively (Additional file 2: Table S2). Except for IAC80 ricinine levels showed an increase in cotyledons was observed at 35°C, while in roots it decreased (Figure 5). Some tocopherols were detected in both root and cotyledon samples and higher levels were detected in the seedlings grown at 35°C (Additional file 2: Table S2). Phytosterols are steroid compounds similar to cholesterol which naturally occur in plants and play important roles in plant adaptation to temperature. Plant sterols are also involved in the regulation of temperature-driven membrane dynamics [35]. In this study, several phytosterols were affected by the temperature. Levels of these compounds were more affected in roots compared to cotyledons. Campesterol, squalene and stigmasta-5,22-dien-3-ol levels increased at 35°C, while *β*-sitosterol decreased (Additional file 2: Table S2).

### Phenotypic and biochemical strategies of different genotypes to cope with the increase temperature

Genotypes MPA11 and MPB01 were developed by the breeding program of the *Empresa Baiana de Desenvolvimento Agrícola S.A* (EBDA-Brazil), while genotype IAC80 was developed by the breeding program of the Campinas Agronomic Institute. All three genotypes were produced aiming to recommend it to small family farms. MPB01 is a relatively small tree (reaching up to 1 m), has a short flowering time (49 days) and produces high yields of seed (2500 kg/ha). MPA11 is 1.30 m high on average, displays a longer flowering time (96 days) and produces lower yields of seed (1900 kg/ha). IAC80 can reach up to 3 m high, has a long life cycle (240 days) and its yield can vary from 1.500 to 4.000 kg/ha. Genotype MPA11 could be clearly differentiated, both phenotypically and biochemically, from genotypes MPB01 and IAC80. Biomass production of genotype MPA11 was far higher than for the other two genotypes at all temperatures. This phenotypic observation was confirmed at the biochemical level. The metabolomes of the genotypes MPB01 and IAC80 were more similar to each other than to the metabolome of MPA11 (Figure 2). Little variation on the metabolite profile due to genotypic differences was observed between genotypes MPB01 and IAC80. Only four metabolites varied significantly between these two genotypes: malate, N-acetylglutamate, sucrose and leucine. In the cotyledons, leucine levels at 20°C were higher for genotype MPB01 than for genotype IAC80, while no differences were observed at 35°C. In the roots, sucrose levels at 20°C were higher for genotype MPB01 than for genotype IAC80, and at 35°C N-acetylglutamate increased and malate decreased in the genotype MPB01 (Additional file 1: Table S1 and Additional file 4: Table S3).

Comparisons between genotype MPA11 and genotypes MPB01 and IAC80 revealed considerably higher significant variation. The levels of some amino acids were reduced in root samples of genotype MPA11 while the levels of most of the identified organic acids increased in the cotyledon samples when compared to the other two genotypes. Thus, increased levels of organic acids in the cotyledons, especially those belonging to the TCA cycle, and reduced levels of amino acids in the roots seem to be an important trait for faster and more efficient growth of genotype MPA11 (Additional file 1: Table S1 and Additional file 4: Table S3).

## Discussion

### Growth and biomass production are strongly affected by an increase in temperature in *R. communis* seedlings

So far, metabolite profiling studies in *R. communis* are limited to two studies describing the use of LC-MS, HPLC-UV and ^1^H NMR-based methodologies to reveal differences in the seed metabolome that could be used to characterize both provenance and cultivar [36],[37]. In the first study, the strongest contributions to the differences in cultivar and provenance were associated with sucrose, phenylalanine, ricinine, *N*-demethyl and *O*-demethyl ricinine [37]. In the second study, the ratio between ricinine and *N*-demethyl or *O*-demethyl ricinine was found to be important for specimen determination [36]. The effect of temperature on plant growth, especially under stress conditions, has been extensively studied [14],[18],[25],[35],[38]. However, there is still a lack of studies that correlate physiological events, such as germination or seedling establishment, with metabolic changes under different environmental conditions. We hereby present a first large scale metabolite profiling study in *R. communis* genotypes that were produced by breeding programs as temperature/drought resistant varieties for family farmers of the semi-arid region of Brazil. This report adds new insights to the understanding of biomass allocation and adaptation to different temperatures during seedling establishment.

*Ricinus communis* showed high plasticity in response to changes of the environmental conditions during initial vegetative growth. It appears that an increasing temperature leads to preferred growth of the stem and true leaves, at the expense of cotyledons and roots. Consequently, a reduction of the root to shoot ratio was observed, which indicates that seedlings are experiencing improved growing conditions. This reduction is also observed as a result of favorable weather, fertilization, irrigation, aeration, or pest control [39]. This growth preference is correlated with a shift from carbon to nitrogen metabolism.

Under drought stress conditions, plants usually produces more root biomass, increasing their root to shoot ratio, as an important trait to acquire drought tolerance [40]. In the light of a usual concomitance of drought with increased temperature, it is surprising that *R. communis* does not invest in its root system in our experiments, unless root growth is not stimulated by high temperature but predominantly by drought. This would be in agreement with the fact that under the prevailing (laboratory) growth conditions there was no drought.

### *R. communis* seedlings adjust their metabolism in order to support growth at elevated temperature, making this species a good candidate for crop production in the lowland tropics

A common feature shared by several temperature-responsive metabolite profiling studies to date is the fact that carbohydrate and amino acid metabolisms seem to be key responsive elements of plasticity and tolerance mechanisms [17]-[20],[25],[27],[41]. Carbohydrates may act as nutrients, osmotic regulators, or signalling molecules through their interaction with phytohormonal networks [42]. Both heat shock and cold acclimation lead to a coordinated increase in the content of amino acids, TCA intermediates (fumarate and malate), amine-containing metabolites (*β*-alanine, GABA, and putrescine) and some carbohydrates, such as maltose, sucrose, raffinose, galactinol and myoinositol [18],[20]. Therefore, an indistinct and unidirectional increase in the content of most of the identified metabolites seems to be the main response to a variety of environmental stimuli. Additionally, temperature shock-responsive metabolites did not seem to be specific to one particular phase in the development of acquired tolerance [18].

**Figure 6:**
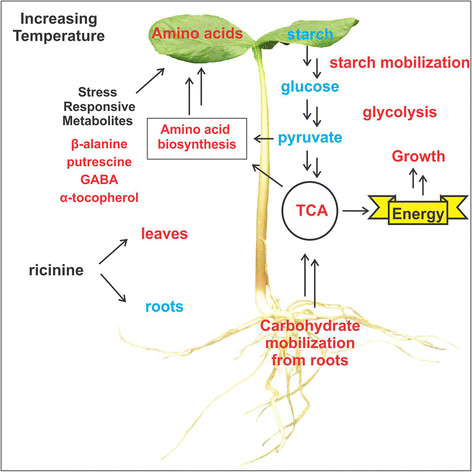
**Schematic changes in*Ricinus communis*seedlings in response to an increasing temperature.** Compounds highlighted in blue show a decrease in response to an increased temperature, while compounds highlighted in red had increased. Pathways highlighted in red are assumed to be up-regulated by the increasing temperature.

*Ricinus communis* showed high plasticity during initial vegetative growth, which was also reflected in the metabolome of the seedlings. An increase in the temperature did not lead to an indiscriminate accumulation of the identified metabolites. Instead, a shift in their carbon-nitrogen balance was observed, in order to adjust development and growth at higher temperature. In *R. communis* seedlings carbohydrate levels were reduced in response to an increasing temperature in both roots and cotyledons. Starch is produced by most green plants as an energy storage compound, which is degraded to produce maltose and glucose by *β*-amylases (BAM) and disproportionating enzyme (DPE1) in the chloroplast [43],[44]. The reduction of starch levels in response to an increase in the temperature suggests that carbohydrate catabolism is triggered when seedlings are experiencing higher temperatures. Starch catabolism-derived signaling has been associated with the juvenile-to-adult phase transition during normal growth and development [42]. Glucose is a key metabolite of many plants, animals, and microorganisms, as an extraordinarily versatile precursor, capable of supplying a vast array of metabolic intermediates for biosynthetic reactions. Glucose, glucose-6-phosphate (G6P), fructose-6-phosphate (F6P) and pyruvate levels are reduced in response to the increase in the temperature indicating that downstream reactions in glycolytic pathway may be up-regulated at 35°C. Transcript levels of several enzymes of the glycolytic and alcohol fermentation pathways were examined in rice seedlings and it was reported that enzymes in this pathway have a sufficient degree of flexibility to adjust to increased energy demands and supply of intermediates for acclimatizing to higher temperatures [45]. The observation of a variation in glycolytic and TCA cycle intermediates in this study confirms the strong relationship between glycolysis and the TCA cycle and indicates that, in response to an increase in temperature, *R. communis* seedlings activate catabolic pathways to generate energy through the oxidization of acetate. Taken together, we conclude that an increase in temperature triggers the mobilization of carbohydrates from the roots, which are then transported through the hypocotyl to the aerial parts, leading to preferred growth of the true leaves, apparently at the expense of the roots (Figure 6).

The carbon skeleton backbones used for amino acid biosynthesis in plants are derived from organic acids produced in glycolysis, the oxidative pentose phosphate pathway and the TCA and Calvin cycles. Amino acids were mostly increased in the cotyledons of young *R. communis* seedlings, whereas in the roots just a few amino acids were identified, but decreasing in content, at elevated temperature. Here, we observed that a decrease in shikimate levels is associated with an increase in its amino acid derivatives tryptophan, tyrosine and phenylalanine as a result of an increasing temperature. In plants, these amino acids are precursors for the production of several important compounds such as phytohormones, electron carriers, enzyme cofactors and antioxidants (tocopherols). *R. communis* seedlings seem to shift their metabolic pool from carbohydrates to amino acids as a dominant biochemical response to adjust growth and developmental processes to the higher temperatures and, likely, to maintain cellular homeostasis. Apparently, fluxes of *R. communis* carbohydrate and amino acid metabolic pathways increase as the increase in temperature increase respiration and growth rate. However, not all crops are able to cope with such high temperatures as 35°C or higher [15]. In fact, most crops show a decrease in total dry matter and seed yield when experiencing increased temperatures [38]. This makes *R. communis* a good candidate for crop production in the lowland tropics.

### *R. communis* seedlings prevent oxidative damage by enhancing the biosynthesis of osmoprotectants molecules and ricinine with increasing temperature

Raffinose family oligosaccharides (RFO) are known to act as osmoprotectants in plant cells, in which high levels of galactinol and raffinose are associated with increased tolerance to stress and are implicated in membrane protection and radical scavenging [46],[47]. RFO are also produced in response to elevated cellular metabolic rate, which is known to produce reactive oxygen species (ROS) as natural byproducts. ROS are highly reactive causing damage to proteins, lipids, carbohydrates and DNA [48].

Polyols can act as stabilizing macromolecules and as effective hydroxyl radical scavengers, thereby preventing oxidative damage of membranes and enzymes [49]. Galactinol is produced from UDP-galactose and myo-inositol by galactinol synthase. This explains why myo-inositol levels were reduced at 35°C, whereas galactinol content increased.

Alpha-tocopherol levels increased in the cotyledons in response to the temperature treatment, but no variation was found in the roots. Delta- and *β*-tocopherol also showed an increase, but to a lesser extent. Tocopherols have many biological functions of which their antioxidant function is the most important and best known. It is generally assumed that increases of α-tocopherol contribute to protect the plant against oxidative damages [50]. Taken together these results suggest that raffinose, galactinol and tocopherols may function to protect *R. communis* cells from oxidative damage caused by the increase in temperature.

Ricinine is an alkaloid with an α-pyridone ring found in Castor bean seeds, seedlings and yellow cotyledons [51],[52], which is involved in the senescence of cotyledons [53]. In the early stages of senescence, ricinine is degraded to N-demethylricinine and O-demethylricinine [53] and at the end of the senescence neither ricinine nor ricinine derivatives can be detected because they are translocated to young healthy growing tissue via the phloem [49],[50]. Ricinine also plays an important role in defense mechanisms against phloem feeders such as aphids [54]. Apart from a possible contribution to defense mechanisms, ricinine may play a role in engaging or modifying primary metabolism, which may be an important prerequisite for successful seedling establishment and adaptation. Ricinine content in genotype IAC80 showed no variance related to temperature, both in the roots and in the cotyledons. However, for genotypes MPB01 and MPA11 an increase in ricinine content in the cotyledons was observed at 35°C, while in the roots ricinine content decreased at 35°C. Therefore, ricinine levels also reflect differences in the seed/seedling metabolome that may be associated with growth rate under different environmental conditions.

## Conclusion

By employing a GC-TOF-MS metabolite profiling approach this study has clearly shown that carbohydrate and amino acid metabolism are tightly coordinated and that they are strongly affected by the temperature during *R. communis* seedling establishment. Due to an increase in temperature, seedlings trigger carbohydrate catabolic pathways as we can infer from the reduced carbohydrate content observed in both roots and cotyledons. Starch is mobilized providing glucose for further utilization in the glycolytic pathway and TCA cycle. Glucose oxidation through glycolysis provides energy and also precursors for amino acid biosynthesis. Carbohydrates are assumed to be mobilized from the roots, through the hypocotyl, to the aerial parts leading to preferred growth of the true leaves, at the expense of the roots. In this paper we raised the question whether this species may have a metabolic signature that may apply to other crops, as well. Here we report that as a result of an increasing temperature, young *R. communis* seedlings show a shift in their carbon-nitrogen metabolism as the main biochemical response to adjust growth and developmental processes to higher temperatures and likely to maintain cellular homeostasis. These observations are extremely important for crops that experience varying environmental conditions during initial vegetative growth, because most crops are not able to cope with temperatures of 35°C or higher, which normally leads to a reduction of biomass production and yield.

Although all three genotypes showed similar responses to the temperatures, we showed that increased levels of organic acids in the cotyledons and reduced levels of amino acids in the roots might contribute to faster and more efficient growth of genotype MPA11. These findings provide insight into the mechanisms underlying temperature tolerance and adaptation during seedling establishment. Furthermore, the metabolic changes observed in response to the increasing temperature provide leads into plant adaptation to varying environmental conditions, which would be very helpful in developing strategies for *R. communis* crop improvement research.

## Competing interests

The authors declare that they have no competing interests.

## Authors’ contributions

PRR carried out the physiological and metabolite profiling experiments, data processing, statistical analysis and draft of the manuscript. LGF, RDC, WL and HWMH participated in the design of the study, coordination and critical reading of the manuscript. All authors read and approved the final manuscript.

## Additional files

## Supplementary Material

Additional file 1: Table S1. Primary metabolites in *R. communis* cotyledon and root samples. Click here for file

Additional file 2: Table S2. Secondary metabolites in *R. communis* cotyledon and root samples. Click here for file

Additional file 3: Figure S1. Redundancy analysis based on polar metabolite profiles in response to an increase in temperature. The distance between genotypes approximates the average dissimilarity of metabolite composition between the two sample classes being compared as measured by their Euclidean distance, whereas the distance between replicates approximates the dissimilarity of their metabolite content as measured by their Euclidean distance. The distance of selected sample symbol (circles) from temperature and genotypes symbols (triangles) predicts the sample membership in one of the classes. It can also be seen as the dissimilarity between metabolite composition of that sample and average metabolite composition of samples belonging to individual classes. Score scaling is focused on standardized metabolites scores. Click here for file

Additional file 4: Table S3. Comparison of metabolite content in different *R. communis* genotypes. Click here for file

Additional file 5: Figure S2. Redundancy analysis based on polar metabolite profiles in response to an increase in temperature. Summarizes the variation in metabolite content that is explained by the increase in temperature and differences between genotypes in the (a) leaves and (b) roots. Click here for file

Additional file 6: Table S4. Significance of metabolite variation in cotyledons and roots of *R. communis.* Click here for file

Additional file 7: Figure S3. Hierarchical cluster analysis in root samples. Heatmap representation of the metabolite-metabolite correlations in response to the temperature treatment in root samples of three *R. communis* genotypes. Correlations coefficients were calculated based on Pearson’s correlation. Click here for file
