# Supplementary figures and images for: Physiological and biochemical responses of Ricinus communis seedlings to different temperatures: a metabolomics approach
Source: BMC Plant Biol. 2014 Aug 12;14:223. doi: 10.1186/s12870-014-0223-5 (PMC4236761; doi:10.1186/s12870-014-0223-5)

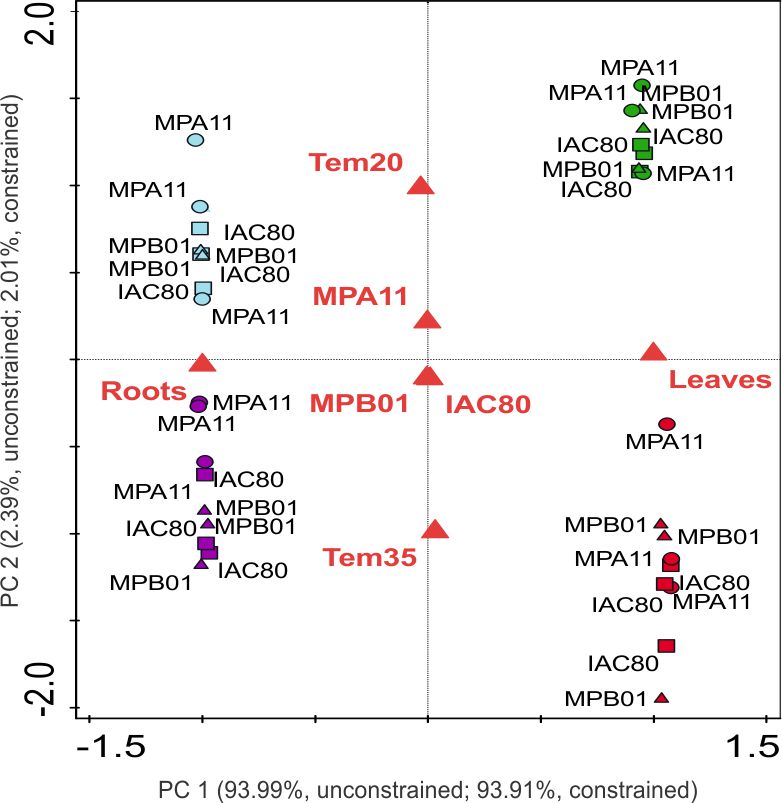

Supplement: Additional file 3: Figure S1. — Redundancy analysis based on polar metabolite profiles in response to an increase in temperature. The distance between genotypes approximates the average dissimilarity of metabolite composition between the two sample classes being compared as measured by their Euclidean distance, whereas the distance between replicates approximates the dissimilarity of their metabolite content as measured by their Euclidean distance. The distance of selected sample symbol (circles) from temperature and genotypes symbols (triangles) predicts the sample membership in one of the classes. It can also be seen as the dissimilarity between metabolite composition of that sample and average metabolite composition of samples belonging to individual classes. Score scaling is focused on standardized metabolites scores. [file s12870-014-0223-5-S3.jpeg]

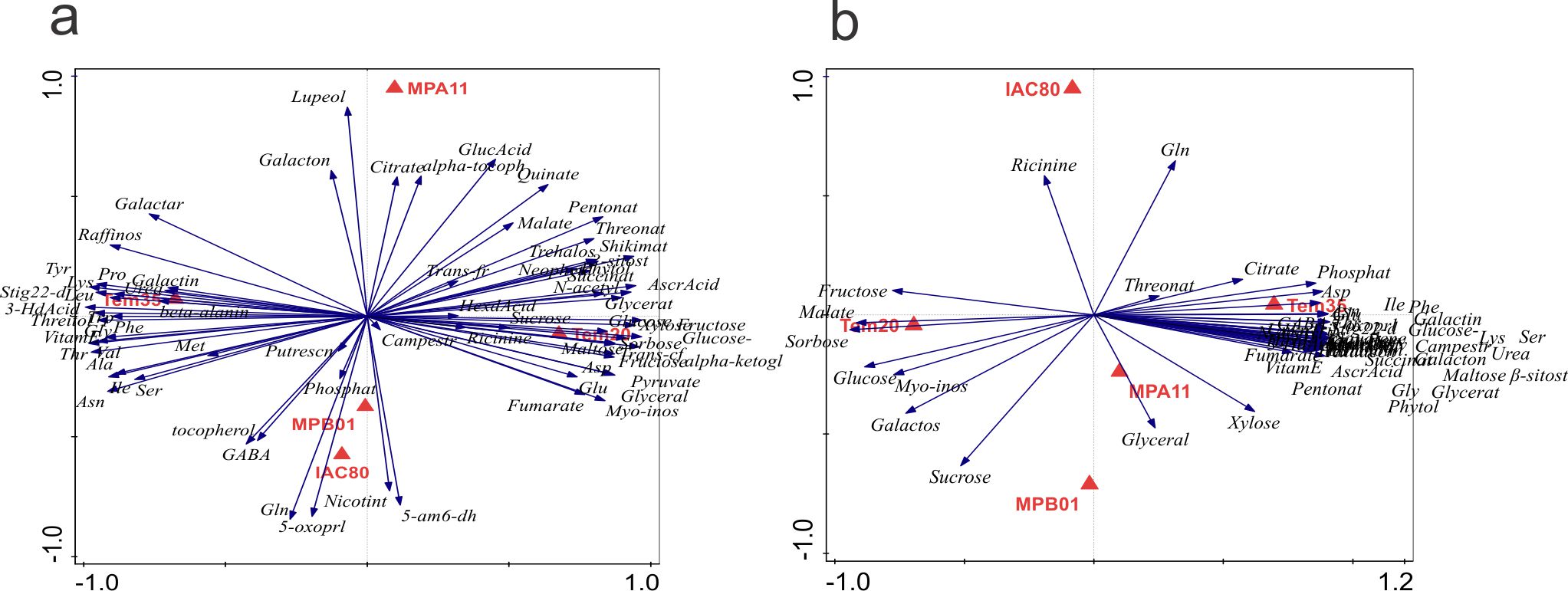

Supplement: Additional file 5: Figure S2. — Redundancy analysis based on polar metabolite profiles in response to an increase in temperature. Summarizes the variation in metabolite content that is explained by the increase in temperature and differences between genotypes in the (a) leaves and (b) roots. [file s12870-014-0223-5-S5.jpeg]

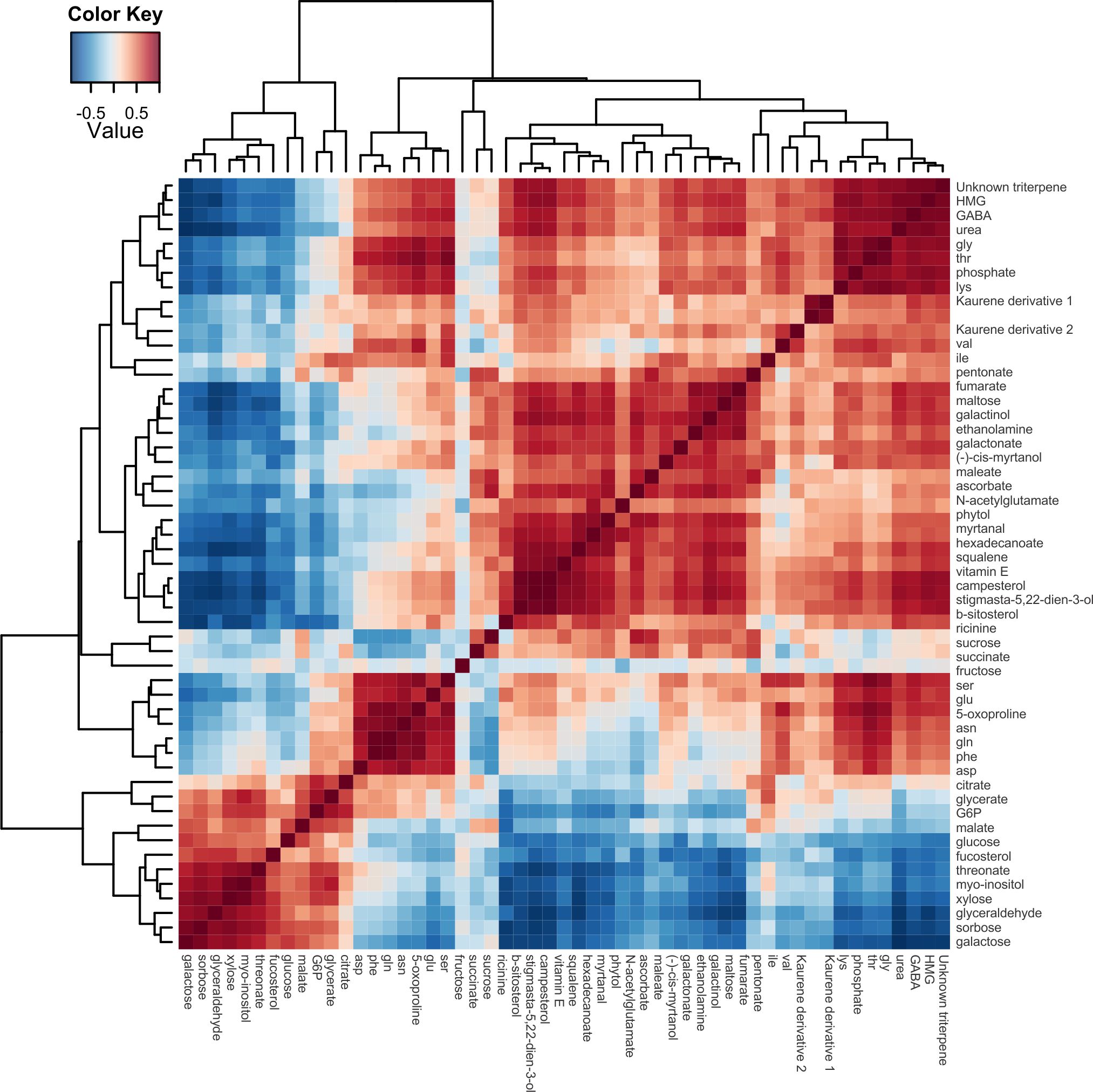

Supplement: Additional file 7: Figure S3. — Hierarchical cluster analysis in root samples. Heatmap representation of the metabolite-metabolite correlations in response to the temperature treatment in root samples of three R. communis genotypes. Correlations coefficients were calculated based on Pearson’s correlation. [file s12870-014-0223-5-S7.jpeg]
